# Effect of parental rearing styles on adolescent ego identity: the mediating role of involutionary attitudes

**DOI:** 10.3389/fpsyg.2023.1292718

**Published:** 2024-01-31

**Authors:** Yan Ding, Changan Sun, Bo Dong

**Affiliations:** ^1^School of Education, Suzhou University of Science and Technology, Suzhou, China; ^2^Qianhuang Experimental Senior High School, Changzhou, China

**Keywords:** adolescent, involution, overcompetitive attitude, parental rearing styles, ego identity

## Abstract

Previous studies have found that negative parental rearing styles can negatively predict the acquisition of ego identity, while it has not been discussed whether the overcompetitive attitudes, a stable personality, will further hinder their ego identity development under the model of educational involutionary. The study used the Overcompetitive Attitude Scale, the Brief Parental Rearing Styles Questionnaire, and the Ego Identity Status Scale to investigate 550 young students in a school in Suzhou in order to explore the influence of parental rearing styles on adolescents’ ego identity development and the role of involutional attitudes. The results showed that: (1) Adolescents’ overcompetitive attitude was positively predicted by parental rejection and overprotection, while it was negatively predicted by parental emotional warmth. (2) Parental emotional warmth significantly predicted adolescents’ ego identity status more favorably than parental rejection, overprotection, and overcompetitive attitude. (3) Overcompetitive attitude plays a partial intermediary role between parental rearing style and ego identity.

## Introduction

In July 2021, with the implementation of the “double reduction “policy, various regions have successively launched the “double reduction “work and achieved positive results. This policy has extraordinary significance for the entire basic education community and even the society. However, due to the imbalance of educational resources, school education is also difficult to meet the needs of students’ heterogeneous learning. The term “involution” has been used to describe or explain contemporary issues in education in recent years. It is defined as the conundrum of increasing internal consumption costs without comparable rewards——Despite spending more on education, parents’ anxiety levels are rising. Children’s schoolwork is getting busier and the exams are getting more complicated (Long and Zhao, 2022). More and more elaborate examinations and score-oriented evaluation mechanisms not only increase the difficulty of learning competition, but also constrain the development of students’ subjectivity and literacy (Xu and Ma, 2021). In the involutional environment, parents’ high expectations and strict requirements force children to bear greater academic pressure. Therefore, parenting anxiety has become an important factor in aggravating involution. The prisoner’s dilemma leads to the transition from academic competition to excessive competition, which not only reflects the alienation of the essence of education by excessive competition, but also has a negative impact on the physical and mental health of young people. Intense learning competition and parental anxiety are the external manifestations of educational involution, and the psychological and behavioral problems of adolescents are the internal manifestations of involution.

According to research, a fiercely competitive learning atmosphere is detrimental to students’ non-cognitive skills (Ito-Morales and Morales-Cabezas, 2021). This may make unpleasant feelings like despair and anxiety worse, which could impede the growth of the body and mind (Posselt, 2021). Through continual competing comparisons, students are more prone to lose confidence and even develop learned helplessness, which lowers their willingness to engage in self-exploration and invest in the future (Gao, 2020). The majority of parents are instigators and implementers of involution, using “reward feedback mechanisms” to maintain their children’s involutional educational model and adopting an educational strategy that caters to their children’s academic competitiveness. This results in the development of unfavorable competitive attitude (Zhang, 2022). Teenagers are subject to the impact and influence of psychological pressure from the learning competition environment while accepting social norms. Psychological crisis is easy to make them confused and at a loss. However, parents are eager to see their children grow up, and their anxiety is gradually transmitted to their children through negative parenting styles and poor parent–child communication relationships. Undoubtedly, it has once again increased the psychological burden of students and hindered their self-identity development and the formation of positive personality traits. As a result, this study aims to shed more light on the relationship between parental rearing styles and adolescents’ ego identity development by examining the mediating role of overcompetitive attitude. This will help us better understand whether the overcompetitive attitude formed by people in the mode of educational involution as a stable personality tendency will further impede their ego identity development.

### Parental rearing styles and adolescent ego identity

Parental emotional support, as an important psychological resource for children, greatly affects the individual’s self-confidence in the face of stress and their ability to solve problems, prompting individuals to explore themselves and challenge difficulties (Sheldon and Epstein, 2005). Negative parenting styles such as parental control and excessive preference will cause individuals to produce more negative self-concepts, which in turn will lead to unreasonable evaluation and cognition of themselves (Aldhafri, 2011; Hughes, 2011; Molina, 2015). The social construction model of resilience holds that resilience is a quality of ability formed with the process of individual self-identity construction (Ungar and Theron, 2020). If self-identity is well constructed, individuals will form a high level of psychological resilience, so that the dilemma can be solved. Yu and Wang (2019) also pointed out that in the middle school stage, the focus of mental health education is to help teenagers master the skills of self-cognition and emotional management, form good will quality, and establish a positive attitude and healthy interpersonal relationship in order to better face setbacks (Yu and Wang, 2019). Therefore, the establishment of self-identity is the most important psychological development task in the adolescent stage. Whether the development of identity is good or not is not only closely related to the individual’s personality improvement and healthy growth, but also related to the individual’s adaptation to social life, the realization of self-worth and the experience of happiness. Individuals with high self-identity will have a clearer understanding of themselves and a stronger sense of self-identity. In the face of the psychological crisis caused by parental anxiety and excessive competition, they can better mobilize psychological resources to cope with it.

According to several studies, the factors that affect the establishment of adolescents’ ego identity mainly include family education, social environment, academic environment, personality characteristics, causal orientation, etc. (An, 2007; Wang and Tong, 2018), of which parental rearing style is one of the most important factors (Masud et al., 2015). Parents are the first socializing agent responsible for transmitting the values, beliefs, and attitudes that will shape the personality of adult children (Palacios et al., 2022). In view of the relationship and interaction between parents and children, and the influence of family environment on children, Darling and Steinberg proposed that parenting styles include the influence of parents on children’s emotional expression and behavior, as well as family atmosphere and family rules with cross-situational stability (Darling and Steinberg, 1993). Prevatt pointed out that parenting style is composed of relatively stable parenting attitudes and beliefs, which reflect parents’ values and behavioral expectations for their children (Prevatt, 2003). Research shows that parents’ active participation in parenting can protect their children from adaptive difficulties and problematic behaviors (Omari and Papaleontiou-Louca, 2020), and harmonious parent–child relationship can provide emotional support for individuals seeking self-development, and promote the adaptive development of adolescent identity (Wang et al., 2017; Moor et al., 2019). However, bad parenting styles, such as parents’ refusal, will affect children’s self-differentiation, which will make them sensitive to setbacks, reduce their level of self-acceptance, and hinder their academic and ego identity development (Cong et al., 2022); Over-protection or psychological control by parents can easily make children weak in character, hinder the development of children’s self-esteem, and form negative self-concept and problem behaviors (Chang et al., 2022). Individuals’ sense of identity and self-worth will be diminished if individuals cannot get enough warmth from their parents’ attitudes (Yao et al., 2022). It can be seen that family warmth and support are the key to teenagers’ psychological growth and personality development. At present, there is no lack of research on parental rearing styles and ego identity, however, it is more practical to explore based on the specific cultural background of “education involution’’.

### Parental rearing styles, overcompetitive attitude, and adolescent ego identity

From the perspective of pedagogy, ‘involution’ is a self-blocking state under excessive competition (Lu, 2021), and a fierce zero-sum competition phenomenon in a limited space (Chen and Bao, 2022). Sampson (1988) proposed that excessive competitiveness is caused by the bad social values and external environment of self-made individualism. It is a personality tendency that individuals pay too much attention to winning and losing while ignoring the realization of self-worth in the face of competitive situations. Therefore, in view of the scholars’ definition of involution and related research results, this study uses excessive competitive attitude to quantify.

The study found that the individual’s competitive attitude is closely related to family environment, personality characteristics, economic conditions and other factors, among which parenting style and parent–child communication will have an impact on children’s competitive attitude. Parental rearing styles can affect children’s competitive attitudes through various verbal and non-verbal forms (Li and Yan, 2017). Specifically, excessive parental control and directive education can lead to children’s lack of autonomy and self-confidence, thus showing too cautious and negative in competition; encouraging children to explore, try and praise their efforts and achievements helps to develop a positive competitive attitude. Individuals’ perception of parental emotional support can positively predict their benign competitive attitude (Chen et al., 2012). On the contrary, negative parenting styles such as refusal to deny or severe punishment will make children feel inferior and insecure. They are forced to meet their parents’ expectations or succumb to their parents’ values and indulge in excessive internal friction competition, trying to gain recognition and appreciation by obtaining rankings and rewards. This excessive competitive attitude will not only have a negative impact on the physical and mental health of individuals, but also affect their ability to interact and cooperate with others. In the long run, it may lead to the limitation of personal development in academic and social aspects.

Some studies have also suggested that competition is an indispensable part of the process of constructing self-concept. Through competition, individuals can compare their own abilities and performance with others, so as to more clearly understand their own strengths and weaknesses (Harter and Bukowski, 2012). However, excessive competition may also undermine individuals’ self-satisfaction, leading them to focus too much on external evaluation and ignore their own internal needs and emotional state (Yang et al., 2018). In the process of guiding individuals to participate in competition, we need to balance the relationship between competition and cooperation, and pay attention to the internal psychological needs of individuals to promote their self-worth. Positive competitive attitude was positively correlated with social adaptation (Park and Baek, 2018; Yang and Robinson, 2018), which means that positive competition helps teenagers achieve growth and progress in self-exploration, and interpersonal relationships can also be well developed. Individuals with high over-competition have lower levels of self-esteem (Zhang et al., 2021), which may hinder individuals from fully expressing and exploring themselves and trigger more self-esteem rumination and identity problems (Gardner and Davis, 2013; Yang et al., 2018). Therefore, it is very important to find a balance point in the competition. Educators and parents should cultivate children to form a good sense of competition, encourage children to believe in their own ability, and avoid the negative impact of excessive and vicious competition. In this way, they can better understand themselves and others, better adapt to society and life so as to achieve higher life satisfaction and happiness.

### The current study

Parental caring was found to be positively associated with current self-involvement and future engagement ambitions, while parental rejection and indifference were negatively related to same outcomes (Wu et al., 2014). The study found that competitive attitudes have an impact on the psychological well-being of individuals (Sheng et al., 2015). Overcompetitive attitude negatively predicts individuals’ clarity of self-concept (Yang et al., 2018). This suggests that there is a strong link between overcompetitive attitude and the development of ego identity in adolescents. Competitive attitudes of secondary school students are significantly associated with parental rearing styles (Li and Yan, 2017). The research also revealed that those with a competitive attitude are more likely to enjoy and value cooperation, as well as emotional contact with others. Can this effectively lessen the negative effects of poor parental on the development of their ego identity? Therefore, this study aims to investigate the relationship between the three variables through quantitative research.

## Materials and methods

### Participants

The study was conducted by utilizing the cross-sectional design. The participants of the study were recruited by convenience sampling. The participants consisted of 512 adolescents (246 females, 266 males) attending two schools in Suzhou. The participants’ ages were between 12 and 18 (*M* = 15.46 years, SD = 1.76 years). Of the participants, 92 (18.0%) were 7th graders, 96 (18.8%) were 8th graders, 64 (12.5%) were 9th graders, 92 (18.0%) were 10th graders, 92 (18.0%) were 11th graders, and 76 (14.8%) were 12th graders.

### Materials

#### Competitive Attitudes Scale – China version

The scale was revised by Chen et al. (2003), which consists of two dimensions of 27 items, positive and overcompetitive attitudes. The participants indicated their response on a 5-point scale (1 = ‘*Strongly disagree*’ to 5 = ‘*Strongly agree*’) with higher scores indicating a higher overcompetitive attitude level. A sample item is “The failure of competition makes me feel that the value of being a person is reduced.” Cronbach’s alpha of the overcompetitive scale was found to be 0.71 for this study. The degree of learning involution in this study was captured by the overcompetitive attitude scale.

#### Short form parental style questionnaire (short form-Egna Minnen av. Barndoms Uppfostran, S-EMBU)

It was measured with 21 items on the Short Form Parental Style Questionnaire. The S-EMBU was designed by Arrindell (1983) in 1999 to determine parental rearing styles and revised by Jiang et al. (2010). It was divided into three dimensions: “rejection,” “emotional warmth” and “overprotective.” A sample item is “Father praises me.” It consists of a 4-point Likert-type response scale, where 1 is *Almost never true* and 4 is *Almost always true*, and. Higher scores on this scale indicate more parents’ behavior in a certain dimension. The Cronbach alpha of father’s rejection was 0.93. The Cronbach alpha of mother’s rejection was 0.93. The Cronbach alpha of father’s emotional warmth was 0.92. The Cronbach alpha of mother’s emotional warmth was 0.92. The Cronbach alpha of father’s overprotection was 0.72. The Cronbach’s alpha of maternal overprotection was 0.72, and the internal consistency coefficient of the overall Cronbach’s alpha of the scale in this study was 0.73.

#### Ego Identity Status Scale

The Ego Identity Status Scale, originally created by Jia (1983) and later translated into Chinese by Zhang (2000) from its Japanese version, is a 12-item scale divided into three dimensions: “present self-involvement,” “past crisis” and “desire for future involvement.” A sample item is “I am working hard to achieve my goal.” It consists of a 6-point Likert-type response scale, where 1 is *Completely disagree* and 6 is *Completely agree*. It’s to measure six states of ego identity, including Achieved (A), Achieved-Foreclosure (A-F), Foreclosure (F), Moratorium (M), Moratorium-Diffusion (M-D), and Diffusion (D).Cronbach’s alpha of the overcompetitive scale was found to be 0.72 for this study.

### Procedures

The participants were recruited from two schools in Suzhou during the 2022–2023 academic year. The researcher firstly received permission from the principals of the schools, and signed informed con-sent was obtained from the participants. The participants were informed about the study’s process and aim before the data collection. Then, the instruments of the study were administered to the students by the researcher during school hours in their classrooms. Participation was voluntary and anonymous, and the participants were independent on withdrawing from the current study at any time. The researcher explained how they could fill out the surveys. The participants completed the surveys of the research in approximately 15 min.

### Data analysis

The data of the present study were analyzed by programs of SPSS 25.0 and Process 4.0. First, descriptive statistics (skewness, kurtosis, mean, and standard deviation) were examined. Second, correlation coefficients between the investigated variables were explored to capture the relationships. Third, path analyses were carried out to examine whether the relationship between parental rearing styles and the development of adolescents’ ego identity was mediated by overcompetitive attitude, as well as to explore the direct relationships among parental rearing styles, ego identity, and overcompetitive attitude. The bootstrapping mediation technique was used with 5,000 resamples and bias-corrected bootstrap 95% confidence intervals to test the significance of the mediating role (Preacher and Hayes, 2008).

## Results

### Descriptive statistics and correlations

Descriptive statistics were presented in Table 1. The findings of correlations demonstrated that parental rejection, overprotective parental and overcompetitive attitude were positively associated (*r* = 0.14, 0.16, 0.23, 0.11, respectively, *p* < 0.01), while parental emotional warmth was negatively associated with overcompetitive attitude (*r* = −0.11, −0.13, respectively, *p* < 0.05). At the same time, there was a negative relationship between overcompetitive attitude and ego identity status (*r* = −0.17, −0.16, −0.18, respectively, *p* < 0.01). Next, Parental rejection, parental overprotection, and ego identity status dimensions were negatively related (*r* = −0.65, −0.67, −0.32, −0.33, −0.69, −0.70, −0.53, −0.51, −0.28, −0.24, −0.52, −0.49, respectively, *p* < 0.01), while parental emotional warmth was positively related with those dimensions (*r* = 0.72, 0.72, 0.34, 0.42, 0.70, 0.73, respectively, *p* < 0.001).

**Table 1:** Descriptive statistics and correlation analysis of each variable.

Variables	M ± SD	*r*									
		1	2	3	4	5	6	7	8	9	10
Age	15.46 ± 1.76										
1	10.34 ± 4.33	—									
2	11.17 ± 4.50	0.70**	—								
3	18.24 ± 5.17	−0.69**	−0.60**	—							
4	18.37 ± 5.03	−0.58**	−0.73**	0.82**	—						
5	16.89 ± 3.75	0.73**	0.54**	−0.48**	−0.39**	—					
6	19.02 ± 3.73	0.42**	0.70**	−0.35**	−0.45**	0.62**	—				
7	14.97 ± 5.76	−0.65**	−0.67**	0.72**	0.72**	−0.53**	−0.51**	—			
8	14.34 ± 3.11	−0.31**	−0.33**	0.34**	0.42**	−0.28**	−0.24**	0.51**	—		
9	15.20 ± 5.45	−0.65**	−0.69**	0.70**	0.73**	−0.52**	−0.49**	0.87**	0.62**	—	
10	45.44 ± 3.38	0.14**	0.16**	−0.11*	−0.13*	0.23**	0.11**	−0.17**	−0.16**	−0.18**	—

*N* = 512; M, mean; SD, standard deviation; *r*, correlation coefficient. *p < 0.05, **p < 0.01, ***p < 0.001, 1-Father rejection, 2-Mother rejection, 3-Father emotional warmth, 4-Mother emotional warmth, 5-Father overprotection, 6-Mother overprotection; 7-Present self-involvement, 8-Past crisis, 9-Future self-involvement desire; 10-Overcompetitive attitude.

### Examining of the mediating role of overcompetitive attitude

**Figure 1:**
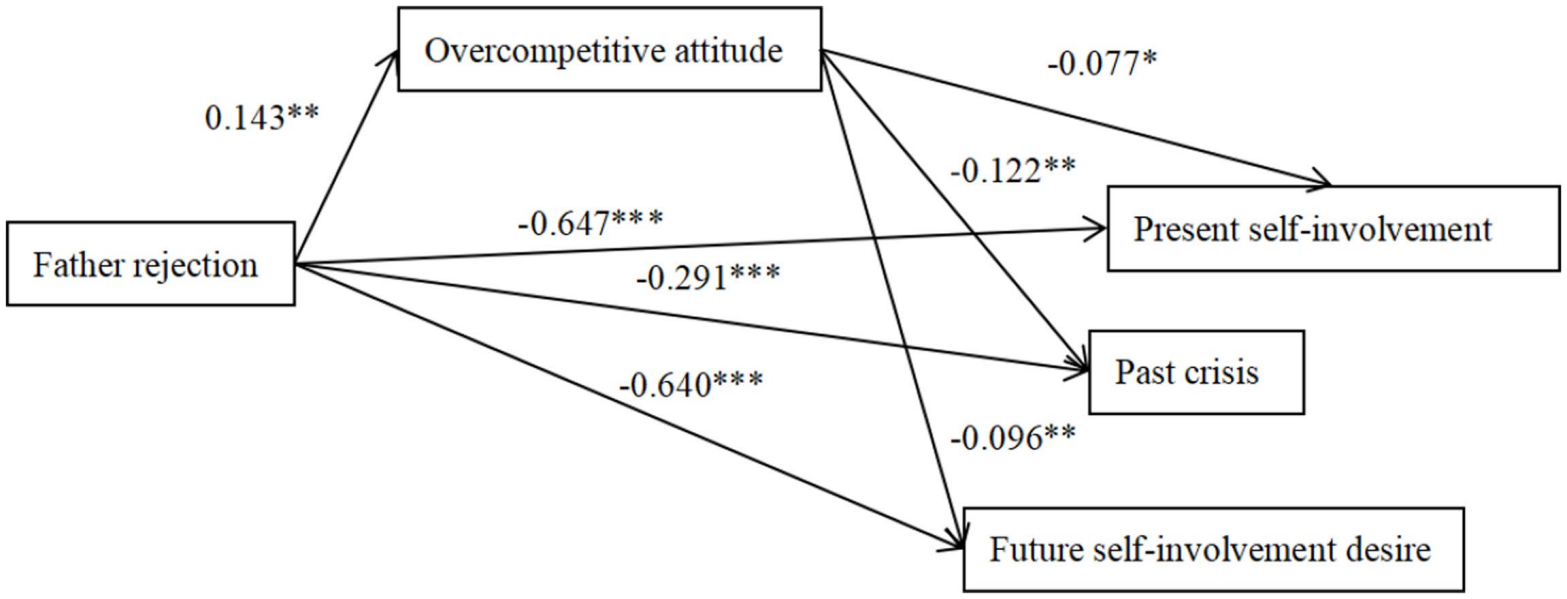
The mediation effect model of overcompetitive attitude between father rejection and ego identity. The factor loadings are standardized. *p < 0.05, **p < 0.01, ***p < 0.001.

**Figure 2:**
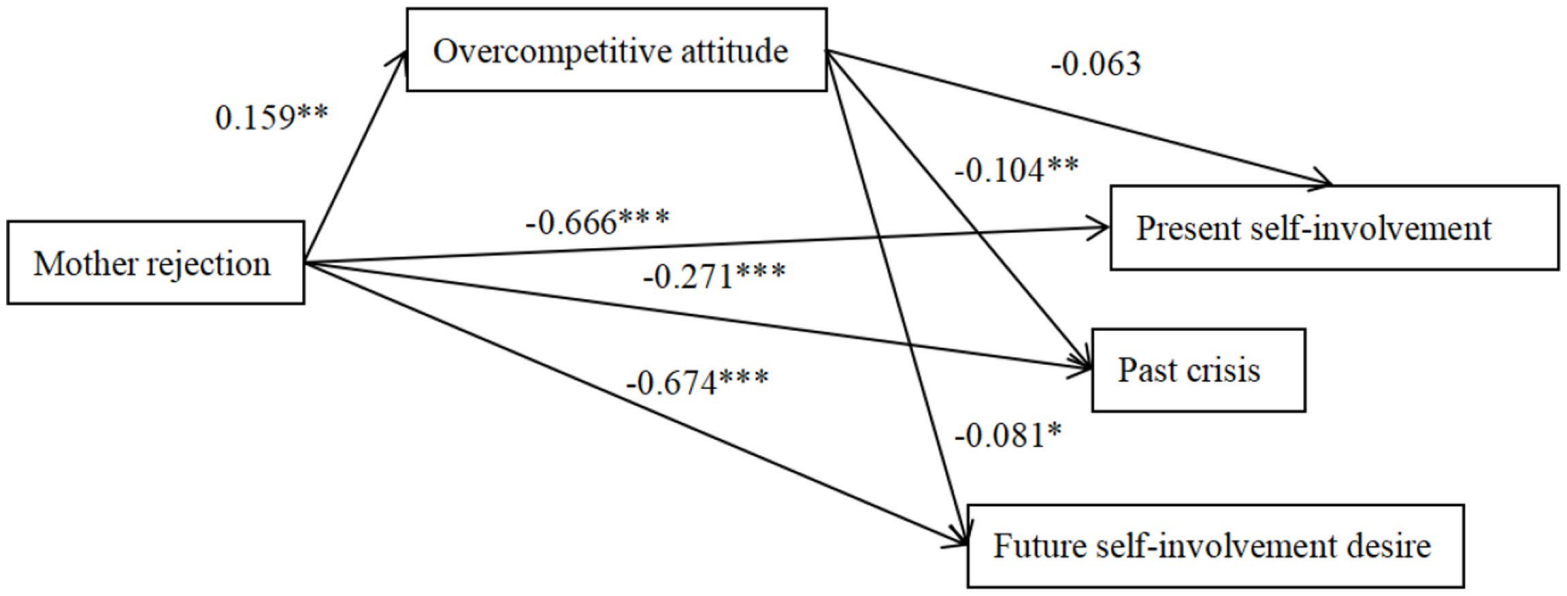
The mediation effect model of overcompetitive attitude between mother rejection and ego identity. The factor loadings are standardized. *p < 0.05, **p < 0.01, ***p < 0.001.

**Figure 3:**
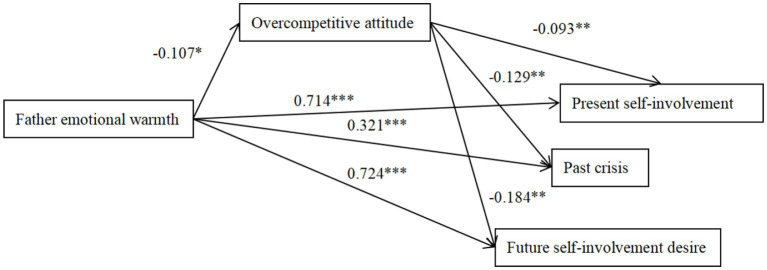
The mediation effect model of overcompetitive attitude between father emotional warmth and ego identity. The factor loadings are standardized. *p < 0.05, **p < 0.01, ***p < 0.001.

**Figure 4:**
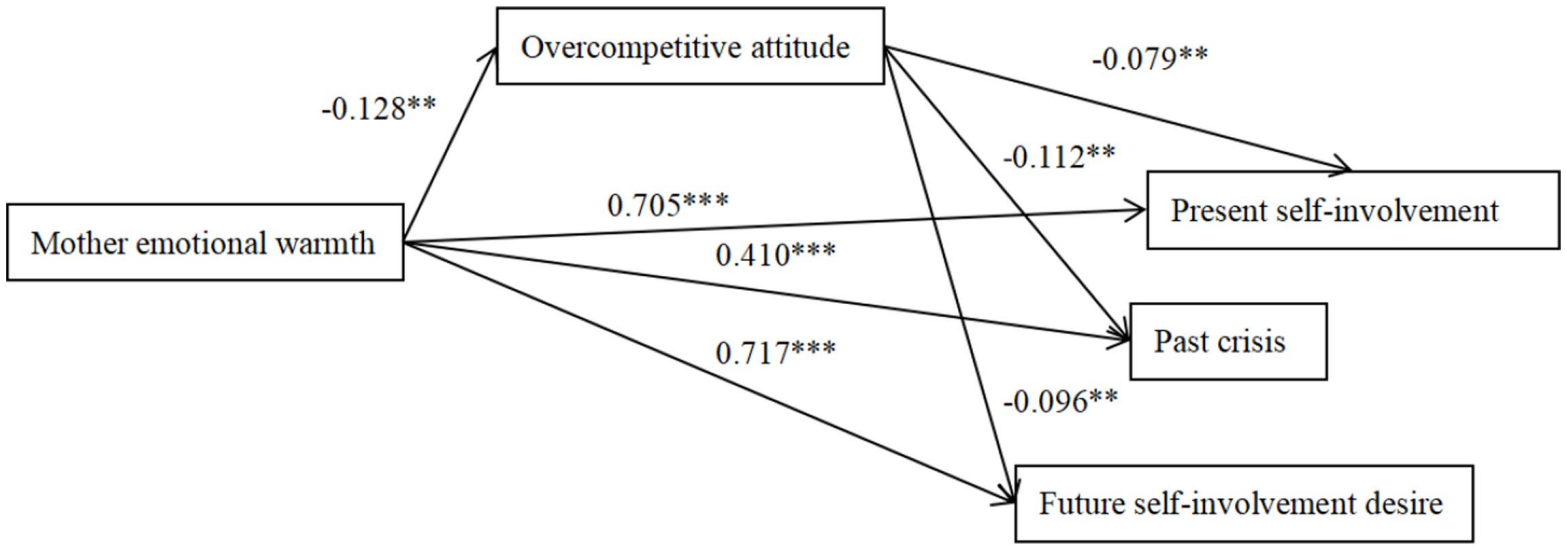
The mediation effect model of overcompetitive attitude between mother emotional warmth and ego identity. The factor loadings are standardized. *p < 0.05, **p < 0.01, ***p < 0.001.

**Figure 5:**
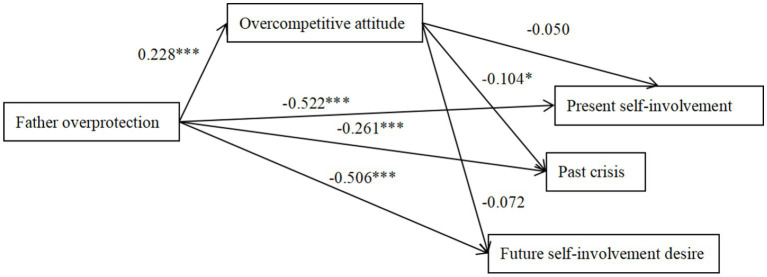
The mediation effect model of overcompetitive attitude between father overprotection and ego identity. The factor loadings are standardized. *p < 0.05, **p < 0.01, ***p < 0.001.

**Figure 6:**
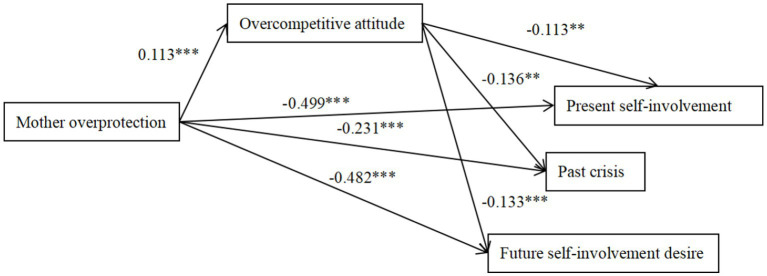
The mediation effect model of overcompetitive attitude between mother overprotection and ego identity. The factor loadings are standardized. *p < 0.05, **p < 0.01, ***p < 0.001.

The path analyses were performed to examine the direct relationships among the investigated variables and the mediating effect of overcompetitive attitude in the total sample (Figures 1–6), the absence of 0 in the interval indicates statistical significance (Erceghurn and Mirosevich, 2008). Parental style was used as a predictor variable, ego identity status as an outcome variable and overcompetitive attitude as a mediating variable. The findings demonstrated that father rejection was related positively to overcompetitive attitude (*β* = 0.143, *p* < 0.01; 95%CI = [0.045, 0.179]) and overcompetitive attitude was related negatively to present self-involvement, past crisis and future desire to invest (*β* = −0.077, *p* < 0.05; *β* = −0.122, *p* < 0.01; *β* = −0.096, *p* < 0.01; 95% CI = [−0.032, −0.002]; 95%CI = [−0.023, −0.004]; 95%CI = [−0.033, −0.005]), indicating that the association between ego identity and father rejection was partially mediated by overcompetitive attitude. Mother rejection positively predicted overcompetitive attitude (*β* = 0.159, *p* < 0.01; 95%CI = [0.055, 0.184]), and overcompetitive attitude negatively predicted past crisis and future desire to commit (*β* = −0.104, *p* < 0.01; *β* = −0.081, *p* < 0.05; 95%CI = [−0.024, −0.003]; 95%CI = [−0.030, −0.005]), indicating that the association between ego identity and mother rejection was partially mediated by overcompetitive attitude.

Father emotional warmth negatively predicted overcompetitive attitude (*β* = −0.107, *p* < 0.05; 95%CI = [−0.127, −0.014])and overcompetitive attitude negatively predicted present self-involvement, past crisis and future desire for input (*β* = −0.093, *p* < 0.01; *β* = −0.129, *p* < 0.01; *β* = −0.184, *p* < 0.01; 95%CI = [0.002, 0.025]; 95%CI = [0.002, 0.017]; 95%CI = [0.003, 0.026]), indicating that the association between ego identity and father’s emotional warmth was partially mediated by overcompetitive attitude. Mother emotional warmth negatively predicted overcompetitive attitude (*β* = −0.128, *p* < 0.01; 95%CI = [−0.144, −0.028]), and overcompetitive attitude negatively predicted present self-involvement, past crisis, and future desire for involvement (*β* = −0.079, *p* < 0.01; *β* = −0.112, *p* < 0.01; *β* = −0.096, *p* < 0.01; 95%CI = [0.002, 0.026]; 95%CI = [0.002, 0.018]; 95%CI = [0.004, 0.025]), indicating that the association between ego identity and mother’s emotional warmth was partially mediated by overcompetitive attitude.

Father overprotection positively predicted overcompetitive attitude (*β* = 0.143, *p* < 0.01; 95%CI = [0.129, 0.282]) and overcompetitive attitude negatively predicted past crises (*β* = −0.104, *p* < 0.05; 95%CI = [−0.037, −0.005]), indicating that the association between ego identity and father overprotection was partially mediated by overcompetitive attitude. Mother overprotection positively predicted overcompetitive attitude (*β* = 0.113, *p* < 0.01; 95%CI = [0.024, 0.181]), and overcompetitive attitude negatively predicted present self-involvement, past crisis and future desire for input (*β* = −0.113, *p* < 0.01; *β* = −0.136, *p* < 0.01; *β* = −0.133, *p* < 0.001; 95%CI = [−0.042, −0.004]; 95%CI = [−0.025, −0.003]; 95%CI = [−0.042, −0.006]), indicating that the association between ego identity and mother overprotection was partially mediated by overcompetitive attitude.

## Discussion

Ecosystem theory suggests that the family environment is crucial for individuals’ early growth (Rosa et al., 2013), and parents shape their kids’ personalities and social development through their educational pursuits (Shan et al., 2020). Different parenting styles have different predictive effects on individual self-identity and self-expression. Children who are fully cared for and supported by their parents in the growth environment usually have positive energy and desire to explore, and they are more confident and enthusiastic about the future. Even in the face of setbacks, they tend to attribute success to their own efforts and abilities, and attribute failure to external factors. This positive mentality and attribution style can often form a positive reinforcement mechanism to help individuals better adapt to setbacks and failures, reduce negative emotions and frustration, and then enhance their investment in the current task and their desire to explore the future, and be more satisfied with their lives, which is consistent with the research results of Chen (2020). Adolescents perceive good emotional support and family atmosphere, resulting in positive cognition, which contributes to the improvement of individual self-efficacy and the enhancement of psychological resilience (Huang et al., 2020). Although classical studies identify the authoritative parenting style as the best parental strategy, Parents’ understanding, support and warmth can promote their children to achieve a good orientation toward others in terms of cognitive and affective empathy and a good self-evaluation in terms of self-concept (Fuentes et al., 2022). High parental warmth is always positive for greater adjustment and less psychological mal adjustment, which was found to benefit professional self-concept (Alcaide et al., 2023; Villarejo et al., 2024). Self-awareness theory states that such individuals will actively create social support when they are frustrated and take full initiative to cope with psychological crises. This result confirms the theory of family functioning, which states that a scientific and rational parental style plays an important role in childrens’ growth and is conducive to the formation of positive psychological qualities such as high self-esteem, self-confidence and resilience (Li and Gan, 2011).

By comparing the differences in the influence of parenting styles on adolescents’ self-identity in each model, it is found that the total effect value of mother rejection is higher than that of father rejection. First of all, due to role cognition and gender differences, the thinking logic of fathers in dealing with problems is very different from that of mothers. When solving parent–child conflicts, more rational methods will be adopted. Although mothers are emotionally delicate, they sometimes adopt extreme ways to make children feel more rejection and rejection, thus generating a sense of distance and negative emotions. In addition, the father who is unknown and reticent often devotes his energy to work. The mother who is more involved in the upbringing is constantly ‘nagging’ for the children’s good, which leads to the gradual formation of parent–child conflicts and even further intensification, thus having a greater impact on the development of adolescents’ self-identity. Secondly, father’s emotional warmth has a greater impact on adolescents’ self-identity development than mother’s emotional warmth. The possible reason is that in the current family structure of China, more and more fathers are aware of the importance of raising children, and they have already broken the image of ‘strict father’. In addition, with the stereotype of ‘loving mother’, children feel more sensitive to the care from fathers than mothers. Good education from fathers can be a guide for children’s mind. At the same time, the father has the image characteristics of bravery, tenacity and strong sense of responsibility. The children will feel more strength and support, so as to dare to take risks without losing stability, actively explore themselves, and better adapt to the social environment and cope with pressure. This is consistent with the results of Miri and Limor (2018). Father often get along with children can make them easy to form a sense of security and self-esteem in interpersonal relationships, so as to get along with others. The difference in parenting formed by gender roles will subtly affect children’s way of doing things and judgment, so it can make up for the weak links in mother’s education. Therefore, the father’s emotional warmth has a far-reaching impact on the establishment of children’s identity.

The study found that the mean score of the overcompetitive attitude sample was 3.50, which was significantly higher than the median score of 2.5, and that the level of over-competence was at the upper middle level, indicating a high level of current involution in adolescent learning. Structural equation modeling revealed that overcompetitive attitude mediated the relationship between parental style and ego identity, and that overcompetitive attitude was negatively associated with past crises, present self-involvement and future investment aspirations. Psychological research has found that parental psychological control over children and adolescent anxiety are causally related to each other (Cong et al., 2019). Teenagers today are constantly overly focused on the results because they are dealing with the psychological weight of academic pressure, parental expectations, and personal growth. Adolescents with overcompetitive attitude have more negative social life events, while emotional stability and high self-esteem are significantly associated with positive competitive attitude (Xu et al., 2018). When individuals focus on improving themselves during competition, they create a new perception of themselves, are more able to explore their potential, and are more self-aware, all of which contribute to the growth of homogeneity. Whereas in ‘involution’ the experience of self is always linked to winning and losing, and the self is based on comparison with others, the experience of failure in learning leads to a negative view of oneself and prevents further exploration of the ego identity (Crocetti et al., 2016; Gerber et al., 2018).

In the context of learning involution, adolescents are prone to self-doubt and no longer have expectations for the future. Adolescents also gradually become more conscious of themselves and increasingly desire external recognition at the same time. Parental rejection can stunt children’s personality growth and lessen their capacity for positive perception and experience (Khaleque, 2013). It is challenging for children to establish a positive self-concept since they feel that their parents do not appreciate or recognize them (Shrout and Weigel, 2020; Li and Liu, 2021). This eventually results in a diminished sense of self-awareness and self-worth (Nie and Gan, 2017). For the child, this is undoubtedly a negative “setback.” Parental overprotection can result in a lack of autonomy, making it difficult for children to adapt to the environment and develop ego identity. Children are forced by their parents’ expectations or values and fall into excessive internal friction competition, which makes it difficult to establish academic self-concept clarity and objective self-cognition, which is consistent with the research results of Liu et al. (2019) and others. The emotional support provided by parents makes it easier for individuals to be satisfied in terms of sense of value, and pays more attention to the improvement of self-ability, which is conducive to the formation of a benign competitive attitude. They are more willing to cooperate with their peers and actively participate in self-exploration activities with others. Feel the pleasure of self-improvement, even for the failure of competition can make a correct and objective evaluation. Therefore, in order to enable students to successfully adapt to the society, it is crucial for parents to guide their children to develop a correct view of academic competition, with self-improvement and potential development as the goal, and not to focus excessively on winning and losing to the point where they base their self-worth on social comparisons., so as to increase their opportunities to experience and explore various roles, and have a positive expectation for the future to promote their self-identity development.

## Conclusion

The current study provided significant implications. Theoretically, this study of adolescents indicated that the relationship between parental rearing styles and adolescent ego identity was partially mediated by overcompetitive attitude. This result presents a more comprehensive understanding of the development of adolescent ego identity. Practically, this result is promising for more effective interventions aimed at preventing ego identity collusion among adolescents from the perspective of system theory. First, parents should set a good example and establish an equal relationship with their children, change the concept of education, and encourage children to establish a healthy competitive attitude with the support of emotional warmth. Second, schools can focus on developing some courses to strengthen students’ self-exploration, create a relaxed and harmonious learning atmosphere and form educational cooperation with parents. Last but not least, advocating and guiding a positive mainstream value, a society full of positive values can provide stronger support for youth self-growth than a society full of cutthroat competition or self-involvement.

In conclusion, this study that aimed to explore the relationships between parental rearing styles, overcompetitive attitude, and ego identity in adolescents found both significant associations among the examined variables and the mediating role of overcompetitive attitude in the relationship between parental rearing styles and adolescent ego identity. Therefore, the present findings may contribute to the existing literature on adolescent ego identity by exploring factors that support or prevent the development of ego identity, hence identifying viable goal intervention pathways.

## Data availability statement

The raw data supporting the conclusions of this article will be made available by the authors, without undue reservation.

## Ethics statement

The studies involving humans were approved by Academic Ethics Review Committee of Suzhou University of Science and Technology Academic Committee. The studies were conducted in accordance with the local legislation and institutional requirements. Written informed consent for participation in this study was provided by the participants’ legal guardians/next of kin.

## Author contributions

CS and BD: Funding acquisition, Methodology, Project administration, Resources, Supervision, Validation, Conceptualization, Writing – original draft, Writing – review & editing. YD: Data curation, Formal analysis, Visualization, Investigation, Software, Writing – original draft.
